# Resolving Position Ambiguity of IMU-Based Human Pose with a Single RGB Camera

**DOI:** 10.3390/s20195453

**Published:** 2020-09-23

**Authors:** Tomoya Kaichi, Tsubasa Maruyama, Mitsunori Tada, Hideo Saito

**Affiliations:** 1 Graduate School of Science and Technology, Keio University, Yokohama 223-8522, Japan; hs@keio.jp; 2 Human Informatics Research Institute, National Institute of Advanced Industrial Science and Technology, Koto-ku 135-0064, Japan; tbs-maruyama@aist.go.jp (T.M.); m.tada@aist.go.jp (M.T.)

**Keywords:** human pose estimation, inertial measurement units, single view, sensor fusion

## Abstract

Human motion capture (MoCap) plays a key role in healthcare and human–robot collaboration. Some researchers have combined orientation measurements from inertial measurement units (IMUs) and positional inference from cameras to reconstruct the 3D human motion. Their works utilize multiple cameras or depth sensors to localize the human in three dimensions. Such multiple cameras are not always available in our daily life, but just a single camera attached in a smart IP devices has recently been popular. Therefore, we present a 3D pose estimation approach from IMUs and a single camera. In order to resolve the depth ambiguity of the single camera configuration and localize the global position of the subject, we present a constraint which optimizes the foot-ground contact points. The timing and 3D positions of the ground contact are calculated from the acceleration of IMUs on foot and geometric transformation of foot position detected on image, respectively. Since the results of pose estimation is greatly affected by the failure of the detection, we design the image-based constraints to handle the outliers of positional estimates. We evaluated the performance of our approach on public 3D human pose dataset. The experiments demonstrated that the proposed constraints contributed to improve the accuracy of pose estimation in single and multiple camera setting.

## 1. Introduction

Inertial measurement units (IMUs) and RGB cameras are utilized for online human pose estimation in real-world settings. IMUs comprise accelerometers and gyroscopes providing measurements of 3D acceleration and calculated 3D orientation. The acceleration and orientation of the IMU attached to each body segment helps infer human motion [1,2,3]. RGB cameras are the most commonly used optical sensors and offer two-dimensional (2D) visual information of the environment. Recent image-based human pose estimation methods detect joints of the human body on the image that offer a robust 2D human pose [4,5,6,7,8]. Both devices are widely used in various motion analysis applications; however, they have physical limitations. IMUs suffer from measuring translational motion due to the integration-drift problem. The position error accumulates in time to reach a remarkable value if it is not reset or compensated, so IMUs cannot provide accurate 3D joint positions in the global coordinates. For RGB cameras, it remains difficult to obtain 3D human pose in the wild using a single view due to depth ambiguity, i.e., the 3D position of the points projected onto the 2D image are indefinite in the optical axis direction.

To compensate for these limitations, researchers have developed full-body motion capture (MoCap) systems that incorporate information from IMUs and RGB cameras. 3D human posture and position are simultaneously optimized to be consistent with the orientation of the IMUs and the silhouettes or joints obtained through convolutional neural networks (CNN) on the images. They have achieved accurate and stable performance in MoCap, but images from multiple viewpoints are required to localize the 3D human position.

Most measurement environments in the real world consist of a single camera rather than multi-viewpoint cameras. In everyday life, cameras (e.g., cameras for surveillance, care systems in the homes of the elderly, and worker-safety systems in factories) are placed to fully cover the space to be monitored. The optimal camera arrangement is to place a minimal number of cameras so that the area where fields of view overlap is small [9]. Assuming these cameras are utilized to capture human posture for the purposes of health care [10,11] or human–robot collaboration [12], a technique for online MoCap in a single-camera environment is desirable. Moreover, inertial sensors have become affordable, and many studies have analyzed human motion using IMUs [13,14,15]. Recently, IMUs have been embedded in many cellphones and smartwatches, and further spread of IMUs is expected.

In this paper, we present an optimization-based method for online 3D human pose estimation that resolves the positional ambiguity of IMU-based poser with a single camera. Single-camera settings impose two challenges on pose reconstruction: (1) A single-view image cannot constrain the position of the human body in three dimensions due to depth ambiguity, and (2) the results of pose estimation are greatly affected by the failure of image-based constraints, such as outlier detection of the joints. For the first problem, we present 3D positional constraints of ground contact. The timing of the contact is determined from acceleration of IMUs, and the contact position is calculated by back-projecting the 2D foot joints on the image into the floor plane. The joints on the image are detected by a CNN-based method [6]. The proposed objective function is designed to handle the outlier detection of the joint detector, which resolves the second problem.

We experimentally evaluated our method using the public 3D dataset TotalCapture [16], which includes all-synchronized videos, IMU data, and ground-truth human pose. The experiments demonstrated that the cost terms incorporated into our objective function contributed to the accuracy and stability of pose estimation.

## 2. Related Work

### 2.1. IMU-Based Motion Capture

Many approaches for IMU-based MoCap have been proposed over the last decade. Huang et al. regressed the pose parameter of the human model from a small set of IMUs and achieved semi-realtime human pose estimation [1]. However, their method does not provide the global position of the solved human model. Although IMU provides accurate orientation in a high frame rate, it is susceptible to drift in global position. A survey reported that a commercial marker-less motion capture suit composed of 17 IMUs suffers from large positional error [17].

To handle this potential hurdle, von Marcard et al. reconstructed human motion using global optimization [2]. As a result that their method optimizes the pose in all frames simultaneously, it is offline. Another approach focused on human–object contact, which constrains one or more positions the subject touches [3]. This method works well when the contact positions are predefined. However, it accumulates the positional error when the contact positions are determined online. Inspired by the contact constraints on pose reconstruction, our approach utilizes RGB images to compensate for the contact’s position ambiguity.

### 2.2. Image-Based Motion Capture

Improvements of deep neural networks have gained the attention of many researchers in human pose estimation. A recent data-driven method estimates 3D human configuration using only a single RGB camera [18,19,20,21,22]. The image-based 3D posers can be roughly divided into two approaches: estimating the 3D position of keypoints (joints and face landmarks), and inferring the pose parameters of a pre-defined human model. The former approaches do not provide the limbs orientation. The latter estimates the full-body posture including the limbs orientation; however, the literature noted that these framewise estimators are typically trained and evaluated on 3D datasets recorded in constrained and unrealistic environments [23]. On the other hand, the accuracy of 2D pose estimators, which detect human keypoints, has been improved by a number of studies over the last decade [4,5,6,7,8]. Due to its performance stability, we utilized one of the open-source 2D joint detectors [6].

### 2.3. Motion Capture Fusing IMUs and Other Sensors

A line of research on combining IMU and visual information has aimed to achieve full-body MoCap free from positional drift. Images from multi-view cameras are utilized to constrain the subject’s position three-dimensionally [16,24,25,26,27]. The posture and the global position of the subject is optimized by minimizing the difference between the human silhouettes on the images and the solved human model projected onto the images [24]. Other studies have found that joint positions on 2D images obtained by a CNN-based keypoints detector improve the performance of 3D MoCap [16,25]. The above-mentioned IMUs and image fusion approaches optimize the pose parameter of the human model using the silhouettes and keypoints. Recent work estimate the 3D joint position by lifting 2D multi-view keypoints to the 3D space [27]. As a result that it directly infers the joint position, it does not provide the limb’s orientation. Although these approaches are appealing because of their stability and accuracy, at least two viewpoints are required to resolve depth ambiguity and localize the subject.

Researchers have addressed pose estimation combining IMU and single view. Some studies have performed 3D human tracking with IMUs and a single depth sensor, such as Kinect [10,28]. However, the measurement accuracy of Kinect decreases outdoors. The only study that has dealt with 3D MoCap with IMUs and a single RGB camera simultaneously optimizes human pose for a certain period of frames, and the global optimization is processed offline [29]. An offline method uses all frames in a sequence to optimize the human pose of a certain frame in the sequence. Offline methods are used for motion analysis after the movement of the subject, especially in the sports and rehabilitation field. On the other hand, online methods that use current frame and/or previous frames to estimate the human pose can be applied to human–robot interactions and monitoring the subjects for healthcare. To the best of our knowledge, no study addressed online MoCap using IMUs and a single RGB camera.

## 3. Methods

### 3.1. Pose Parameterization and Calibration

We parameterize the subject’s pose using a Digital Human Model (DHM) [30] that consists of a 48 degrees of freedom (DoF) link configuration. The model provides kinematics and the body mesh when the pose including the global translation θ ($\in R^{51}$) is determined. We extend the IMU-based MoCap method [3] for pose parameterization and optimization.

**Figure 1:**
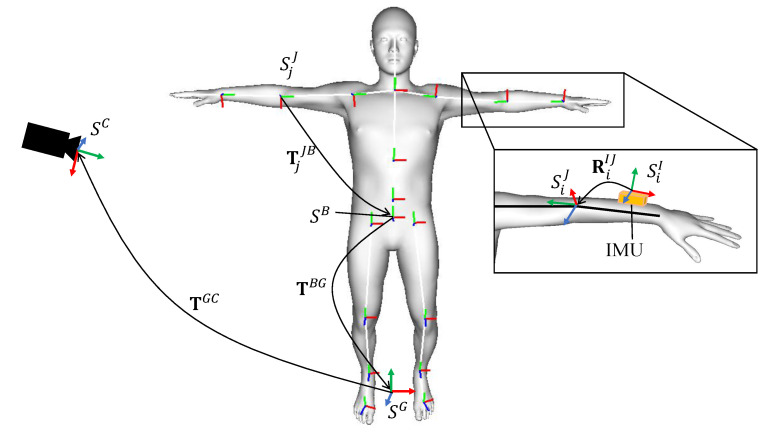
Relations among the local coordinate systems.

The transformation matrices among global coordinates $S^{G}$, camera coordinates $S^{C}$, body coordinates $S^{B}$, *j*-th joint coordinates $S_{j}^{J}$, and *i*-th IMU local coordinates $S_{i}^{I}$ are required for fusing the sensors on motion tracking. Figure 1 shows relations between the coordinates and transformation matrices. The transformations between the global coordinates and the camera coordinates $T^{\mathrm{GC}}$ is determined using a checkerboard [31]. In our configuration, the checkerboard is placed on the floor. The Z-axis of the global coordinates $\left(X_{w},Y_{w},Z_{w}\right)$, defined by the checkerboard, points in the opposite direction of gravity, and the $Z_{w}=0$ plane coincides with the floor. Note that the checkerboard can be removed after the camera is calibrated and fixed. After the camera setup, the subject wearing IMUs takes a calibration pose (e.g., T-pose: standing upright and keeping both arms horizontal). The rotational transformation from each IMU to the joint coordinate is obtained from

(1) $$R_{i}^{\mathrm{IJ}}=R_{i}^{J}\left(\theta_{0}\right)\cdot {\left(R_{i}^{I}\left(t_{0}\right)\right)}^{-1},$$

where $R_{i}^{I}\left(t_{0}\right)$ represents the *i*-th IMU sensor orientation in the global coordinates when the subject takes the calibration pose, and $R_{i}^{J}\left(\theta_{0}\right)$ denotes the rotation matrix of the model joint belonging to the bone to which the IMU is attached in the global coordinates. $t_{0}$ and $\theta_{0}$ represent the frame and pose parameter of the calibration pose, respectively. As illustrated in Figure 1, $R_{i}^{J}\left(\theta_{0}\right)$ can be represented by the conversion of the coordinates from the global coordinates $S^{G}$ to the local coordinates of each joint $S_{j}^{J}$ of the human model. It can be calculated by transformation matrix $T_{j}^{\mathrm{JB}}\left(\theta_{0}\right)$ and $T^{\mathrm{BG}}\left(\theta_{0}\right)$. $T_{j}^{\mathrm{JB}}\left(\theta_{0}\right)$ denotes the transformation from $S_{j}^{J}$ to the body coordinates $S^{B}$. In our method, $S^{B}$ is defined to correspond with the local coordinates of the pelvis joint of the human model. The transformation $T_{j}^{\mathrm{JB}}\left(\theta_{0}\right)$ can be obtained from the forward kinematics of predefined link configuration of the model. $T^{\mathrm{BG}}\left(\theta_{0}\right)$, transformation from the body coordinates to the global coordinates, is determined by the position and orientation of the subject taking the calibration pose.

For synchronizing the data from IMUs and a camera, a physical cue that can be detected from both the camera and IMUs can be used when it is difficult to synchronize a camera and multiple IMUs with a signal synchronizing apparatus. For example, a footstamp is applicable because, for the camera, the timing of the cue is obtained from the motion of ankle joint detected on the image, and for the IMUs, the timing can be calculated from the acceleration measurements of the IMU attached to foot. The synchronization should be performed after the calibration pose.

### 3.2. Full-Body Pose Optimization

We follow the paradigm of constraint-based motion tracking. More specifically, we minimize the following total cost function composed of multiple cost terms on a per-frame basis.

(2) $$E\left(\theta \right)=E_{O}\left(\theta \right)+\lambda_{RoM}E_{RoM}\left(\theta \right)+\lambda_{P}E_{P}\left(\theta \right)+\lambda_{G}E_{G}\left(\theta \right),$$

where $E_{O}\left(\theta \right)$ and $E_{RoM}\left(\theta \right)$ constrain the orientation and the range of motion of the model joints, respectively. $E_{P}\left(\theta \right)$ and $E_{G}\left(\theta \right)$ represent the positional error of the joints and the ground contact points, respectively. We design these positional error terms so as to stably estimate the human pose in an under-constrained environment. Every term is weighted by a corresponding weight λ. The quasi-Newton algorithm [32] is applied to solve the optimization problem.

#### 3.2.1. IMU-Based Constraints

The orientation of the kinematic links is estimated from the measured orientation of IMU sensors. The cost term is represented as the sum of the orientation differences between IMU measured and estimated bone orientation. Here, the *i*-th IMU offers its orientation in each local coordinates. Using the transformation matrix from the sensor coordinates to the joint coordinates $R_{i}^{\mathrm{IJ}}$ (Equation (1)), the cost $E_{O}\left(\theta \right)$ can be expressed as

(3) $$E_{O}\left(\theta \right)=\sum_{i=1}^{N_{I}}{∥R_{i}^{\mathrm{IJ}}\cdot R_{i}^{I}-R_{i}^{J}\left(\theta \right)∥}_{F}^{2},$$

where $R_{i}^{I}$, and $R_{i}^{J}\left(\theta \right)$ denote the sensor measurement and solved value of bone orientation in the current frame, respectively. $N_{I}$ describes the number of IMUs.

The other IMU-based constraint, $E_{RoM}\left(\theta \right)$, adds cost when the estimated joint angle exceeds or falls short of the RoM ψ. ψ defines the minimum and maximum joint angles, i.e., $\psi \in \{\left(\psi_{r}^{\min},\psi_{p}^{\min},\psi_{y}^{\min}\right),\left(\psi_{r}^{\max},\psi_{p}^{\max},\psi_{y}^{\max}\right)\}$, where *r*, *p*, and *y* represent the three principal axes in the joint coordinates. The cost for each joint is calculated according to

(4) $$e_{RoM}\left(\phi \left(\theta \right),\psi \right)=\sum_{k\in \{r,p,y\}}\left\{\begin{array}{cc} \rho ({\left(\phi_{k}\left(\theta \right)-\psi_{k}^{\min}\right)}^{2}) & \left(\phi_{k}\left(\theta \right)<\psi_{k}^{\min}\right)\\ \rho ({\left(\phi_{k}\left(\theta \right)-\psi_{k}^{\max}\right)}^{2}) & \left(\phi_{k}\left(\theta \right)>\psi_{k}^{\max}\right)\\ 0 & \left(otherwise\right) \end{array},\right.$$

where $\phi_{k}\left(\theta \right)$ represents the estimated rotation around the *k*-axis of the joint. ρ(·) is a loss function detailed in Section 3.2.2. Then, we can compute the RoM cost for the entire body by

(5) $$E_{RoM}\left(\theta \right)=\sum_{j=1}^{N_{J}}e_{RoM}\left(\phi^{\left(j\right)}\left(\theta \right),\psi^{\left(j\right)}\right),$$

where $N_{J}$, $\phi^{\left(j\right)}\left(\theta \right)$, and $\psi^{\left(j\right)}$ denote the number of joints whose rotation is estimated, the *j*-th joint angles, and the *j*-th joint RoM, respectively. We adopt the RoM defined in the commercial Digital Human Model [30].

#### 3.2.2. Image-Based Constraints

$E_{P}\left(\theta \right)$ constrains positional differences between keypoints on an image $p^{C}$ detected by a CNN-based 2D pose estimator [6] and corresponding 3D joint positions projected onto the image ${\hat{p}}^{C}$. The 3D point of the solved model in the body coordinates ${\hat{P}}^{B}$ can be projected to the camera coordinates by

(6) $${\hat{p}}^{C}\left(\theta \right)=T^{\mathrm{GC}}T^{\mathrm{BG}}\left(\theta_{0}\right){\hat{P}}^{B}\left(\theta \right),$$

where $P_{j}$ denotes the 4D column vector, which represents the 3D joint position in a homogeneous coordinate system. $T^{\mathrm{GC}}$ and $T^{\mathrm{BG}}\left(\theta_{0}\right)$ are the 4×3 translation matrices described in Section 3.1.

As a result that the global position of the estimated model is constrained by visual information from only one RGB camera, the failure of the 2D joint detector seriously compromises motion tracking accuracy. To improve the robustness to such outlier detection of keypoints, we extend Tukey’s biweight. Specifically, the cost term of a joint is less weighted when the joint-position estimate is far from the model joint in the previous frame. The weight is calculated by

(7) $$w_{p}=\left\{\begin{array}{cc} \exp(-\frac{d_{p}^{2}}{2s^{2}k_{p}^{2}}) & \left(d_{p}\leq \beta_{d}sk_{p}\right)\\ 0 & \left(otherwise\right) \end{array},\right.$$

where $p\;(1\;\leq \;p\;\leq \;N_{P})$, $\beta_{d}$, and *s* are the index of detected joints, a hyperparameter that controls the range of nonzero weight, and the scale of distribution, respectively. Here, $N_{P}=18$, $\beta_{d}=2$, and s=140 in our experiments. $d_{p}$ represents the Euclidean distance between the detector estimate and the projected point of the corresponding joint in the previous frame, and $k_{p}$ denotes the standard deviation of the weight distribution. The distribution of keypoints detected by the data-driven 2D pose estimator depends on the keypoint type. For example, the distribution of an eye must be smaller than that of hips. The value of $k_{p}$ is defined by object keypoint similarity (OKS) [33], which is used to evaluate the performance of the 2D keypoint detectors; that is, keypoint detectors ensure accuracy in this distribution. The positional cost weighted with $w_{p}$ is expressed as

(8) $$E_{P}\left(\theta \right)=\sum_{p=1}^{N_{P}}\rho (w_{p}c_{p}^{\mathrm{im}}∥p_{p}^{C}-{\hat{p}}_{p}^{C}\left(\theta \right){∥}_{F}^{2}),$$

where $c_{p}^{\mathrm{im}}$ represents the confidence score from the keypoint detector.

In our single-camera setting, $E_{P}\left(\theta \right)$ alone cannot localize the global position of the model due to the camera’s depth ambiguity. To optimize the model position three dimensionally, we present the ground contact cost term $E_{G}\left(\theta \right)$. Fusing IMU acceleration and positional measurement from the camera, $E_{G}$ minimizes the distance between foot position and ground contact point.

**Figure 2:**
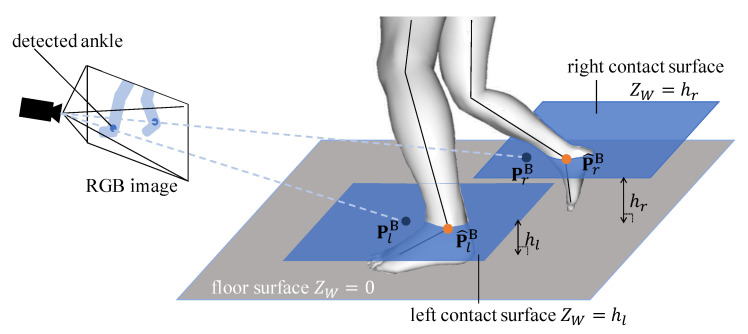
Visualization of the ground contact constraint.

We define the cost as depicted in Figure 2. Let ${\hat{P}}_{g}^{B}\left(\theta \right)$, where g∈{left_foot,right_foot} is the left or right ankle position of the estimated model, and let $P_{g}^{B}$ be the intersection between the contact surface and the line where the 2D ankle keypoint is back-projected into three dimensions. The contact surfaces are the planes parallel to the floor plane, and each contact surface passes through each ankle of the solved model. The floor plane can be determined by camera calibration as described in Section 3.1. The confidence score $c_{g}^{G}$ that the foot is on the ground is determined from the acceleration of the foot-attached IMU and the height of the foot. The resulting ground contact cost is calculated according to

(9) $$\begin{array}{cc} E_{G}\left(\theta \right)= & \;\sum_{g}\rho (c_{g}^{G}w_{g}c_{g}^{\mathrm{im}}∥P_{g}^{B}-{\hat{P}}_{g}^{B}\left(\theta \right){∥}_{F}^{2}),\\ \mathrm{where}\;c_{g}^{G}= & \;\delta +\left\{\begin{array}{cc} \beta_{G}/∥a_{g}∥ & \left(\beta_{G}/∥a_{g}∥\leq 1\right)\\ 1 & \left(otherwise\right) \end{array},\right. \end{array}$$

where $a_{g}$ and $\beta_{G}$ represent the acceleration measured by the IMU attached to the foot *g* and a constant value to determine the gradient, respectively. For all experiments, $\beta_{G}=5$ and $\beta_{G}/∥a∥$ was calculated using $\beta_{G}/\left(∥a∥+\epsilon \right)$, $\epsilon =1.0\times 10^{-6}$ to avoid zero division. δ takes 1 when the lowest mesh of *g* is lower than that of the other foot, and 0 otherwise. $w_{g}$ is also multiplied for handling outlier detection of foot keypoints. In our method, the Cauchy loss function, ρ(x)=log(1+x), is used as a loss function ρ(·) in the range of motion cost term $E_{RoM}$, image-based positional cost term $E_{P}$, and ground contact cost term $E_{G}$. The Cauchy loss function suppresses extremely large values so that the effect of the error of one joint on the total loss does not become too large in the process of the optimization calculation. An example of extremely large error is that when the distance from the camera to the subject is large and camera position is relatively low, the small 2D position error of detected joints on the image causes huge error in the 3D space.

## 4. Evaluation

### 4.1. Dataset

We quantitatively evaluate the performance of our approach on 3D human pose dataset TotalCapture [16]. TotalCapture provides 60 fps of all-synchronized IMU data, HD videos from fixed cameras, and ground-truth human pose measured by optical MoCap. A total of 13 IMUs are attached on the head, sternum, pelvis, upper and lower limbs, and feet. Our method uses acceleration and orientation of IMUs, and an image sequence from a single camera. Note that optical MoCap data are not used for our approach. The original ground-truth of the joint position and orientation is obtained by fitting the marker position measured by optical motion capture system to the surface of the human model. The human model of the optical motion capture has a different definition of the link structure from that of DHM we used for pose estimation. For example, the pelvis joint to neck joint is divided into 5 segments in the original ground-truth, but it is divided into 3 segments in DHM. Therefore, it is not possible to make a strict comparison of the joint position and orientation between the estimated pose of DHM and the original ground truth. Hence, we determined the joint position and orientation of DHM so that the Vicon 57-point markers defined in advance on the DHM surface matches the marker position measured by optical motion capture [30], and used it as the ground-truth in this experiment.

We quantitatively evaluated our method following the standard evaluation protocol defined in [16]. In the protocol, the test set consists of 15 scenes in total including the scenes Walking 2 (W2), Acting 3 (A3), and Freestyle 3 (F3) of Subjects S1, S2, S3, S4, and S5. However, there are several sequences in which both feet are off the ground for several frames in a row, such as jumping, in S2-F3, S3-F3, and S5-A3. These scenes are excluded from our dataset and we used S2-ROM3 (S2-R3), S3-F1, and S5-F1 instead. The limitations on the scenes where our method is effective will be mentioned in Section 5.

### 4.2. Implementation Details

We utilized a human model generated statistically from the height and weight of the subject, which is offered by DHM software [30]. Before starting the pose estimation, the subject took T-pose as a calibration pose. During the calibration pose, the global coordinates $\left(X_{W},Y_{W},Z_{W}\right)$ is defined so that the subject stands on the plane at $Z_{W}=0$. For the model of the 2D joint detector used in image-based constraints, we utilized the weights of public pretrained model [6]. No additional training or finetuning is conducted.

The weighting parameter controls the contribution of each cost term to the overall cost Equation (2). The algorithm based on Tree-structured Parzen Estimator is used to seek the parameter values. Several scenes other than the test set are used for parameter tuning and the value found are $\lambda_{RoM}=0.01$, $\lambda_{P}=5.0\times 10^{-4}$, and $\lambda_{G}=5.0\times 10^{-3}$. The parameters are fixed through all experiments.

### 4.3. Contribution of the Proposed Cost Terms

We evaluated how the proposed cost term $E_{G}\left(\theta \right)$ and the adaptive biweight $w_{p}$ work in the constraint-based pose optimization. In this experiment, a full set of 13 IMUs and a single camera that captures entire movement in the field of view were used. The position error in this section represents the mean 3D Euclidean distance between the estimated model and the ground truth over the 16 joints.

**Figure 3:**
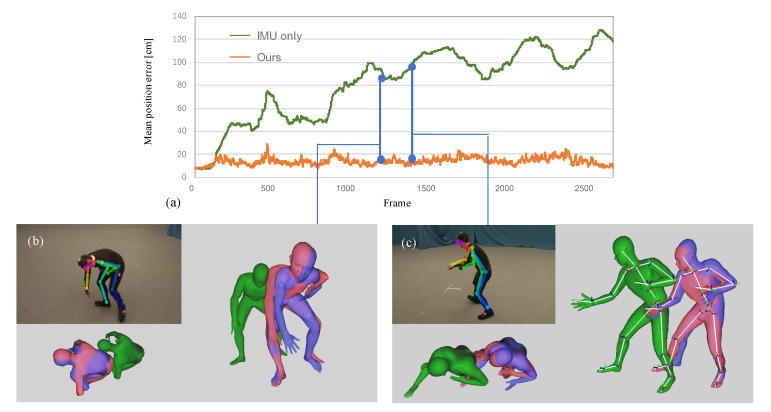
The top graph (**a**) represents per joint mean position error for each frame. The bottom figures (**b**) and (**c**) illustrate the the view of the used single camera and the detected joints by the 2D joint detector, OpenPose [6]. The human models colored in green, red, and blue represent the inference by IMU only, the proposed approach, and ground-truth from optical MoCap, respectively. It is observed that the position of the foot touching the ground is estimated correctly.

The graph of Figure 3a represents per-frame mean Euclidean distance between the solved pose and ground-truth. Figure 3b,c visualize the output of the 2D joint detector [6], and the human models colored in green, red, and blue represent the 3D human pose solved by the IMU only method [3], the proposed method, and optical MoCap (ground-truth), respectively. The estimated 2D joints and 3D models in (b) and (c), respectively capture the same frame in the same scene.

Figure 3a and the human model visualized from above revealed that our approach using a single camera prevented the accumulation of position error. The right foot in (c) is self-occluded and the misdetection occurred; however, our approach robustly optimized the 3D full-body pose. Focusing on the feet in (b) and (c), the foot touching the ground and fixed (right foot in (b) and left foot in (c)) are estimated with higher accuracy in these frames. It would be due to the proposed ground contact cost term.

**Figure 4:**
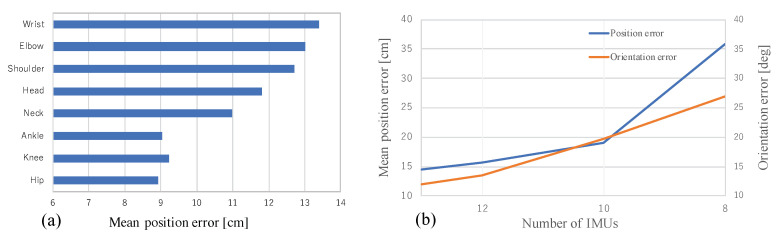
(**a**) Mean per-joint positional error of the human motion capture (MoCap) by the proposed method on all the scenes in the test set. The error values of wrist, elbow, shoulder, ankle, knee, and hip represent the average error of the both side of the segments, i.e., the error of the wrist denotes the average error of left wrist and right wrist. (**b**) Mean 3D position and orientation errors on subjects S3-F1 and S4-F3 with 8 to 13 IMUs.

Table 1 summarizes the quantitative results for pose estimation using the position error metric. RGB only [34] is the state-of-the-art of 3D human pose estimation using only a single RGB camera. $F(E_{O},E_{RoM},E_{P})$ estimates the human pose by minimizing the cost function composed of $E_{O}\left(\theta \right)$, $E_{RoM}\left(\theta \right)$, and $E_{P}\left(\theta \right)$. The results revealed that the ground contact cost term $E_{G}\left(\theta \right)$ improves the positional error. $F(full,w_{p}=1)$ optimizes the pose by Equation (2), but adaptive weight $w_{p}$ is fixed to 1. Meanwhile, the proposed cost function F(full) calculates $w_{p}$ according to Equation (7). Although the mean error of F(full) in the 15 scenes was smallest, $F(full,w_{p}=1)$ estimated the human pose with the highest accuracy in more than half of the test scenes. Especially in Walking 2 (W2), $F(full,w_{p}=1)$ outperformed F(full) in 4 out of 5 trials. The results indicate that in the scene where the 2D joint detector estimates the 2D pose of the subject with high accuracy, the 3D pose reconstruction accuracy is slightly lowered by the adaptive biweight $w_{p}$; however, $w_{p}$ stabilizes the 3D pose estimation when there are misdetections of the joints on a image due to the self-occlusion or unusual posture of the subject (included in Freestyle 3 and Acting 3). The effect of the ground contact cost term is validated from Figure 4a. It represents per-joint position error of human model estimated by the proposed method with a single view and 13 IMUs. Although the estimation error of the hands and feet tends to be large because the limbs move a lot, the positional error of the ankle is relatively small due to the 3D positional constraints of the ground contact.

The mean orientation error of joints is shown in the bottom of Table 1. The error of IMU only and the proposed method (F(full)) were 8.75 degrees and 8.83 degrees, respectively, and no significant differences were observed.

The proposed method can easily be extended to use multi-view cameras by adding the image-based cost function $E_{P}\left(\theta \right)$ and $E_{G}\left(\theta \right)$ for each camera and simultaneously minimize the total cost. We performed the experiments using 8 cameras and 13 IMUs. The state-of-the-art approach for 3D MoCap that infers both joint position and orientation from IMUs and multiple images [25] extracted several images from TotalCapture to test their approach. The performance of our approach was compared with [16,25] on the same scenes as the test set of [25], excluding the scenes where the subject jumped. As shown in Table 2, in several scenes, our method outperformed the conventional approach that optimizes the pose parameter to reconstruct human motion. In the scene where our approach was inferior in accuracy (S2-R3), the subject frequently crouched and bent forward. It appears that these motions caused self-occlusion of the ankle and the ground contact constraint did not work. The experiments demonstrate that the proposed ground contact constraint contributes to improve the accuracy of 3D human pose estimation in multi-view camera setting as well as single-camera setting when the floor plane is pre-defined and the foot can be detected from the camera.

### 4.4. The Number of IMUs

Wearing many IMUs takes time and hampers the subject’s range of motion. Towards the real-world use of our method, we investigated the relation between the accuracy of the pose estimation and the number of IMUs. The experiments were conducted with (1) 13 IMUs: full set as described in Section 4.1, (2) 12 IMUs: full set without head, (3) 10 IMUs: IMUs on upper arms removed from (2), and (4) 8 IMUs: IMUs on upper legs removed from (3). 3D position and orientation errors in different IMU configurations are shown in Figure 4b.

The decrease of the IMUs largely affects the accuracy of both position and orientation. It would be because our single-camera approach does not constrain joint positions other than the foot in three dimensions. In the experiments on IMU only and $F(E_{O},E_{RoM},E_{P})$, the objective function diverged with 8 IMUs. The proposed ground contact cost term $E_{G}\left(\theta \right)$ and $w_{p}$ contributed to the convergence of pose estimation.

## 5. Conclusions and Future Work

We have presented the first online approach to estimate the 3D human pose fusing IMUs and a single camera. In order to constrain the position of the solved model in three dimensions, the proposed cost term detects the timing and position of foot grounding. We handle the outlier of visual information by extending the biweighting algorithm. The experimental results showed that the proposed objective function stably estimated the 3D human pose, including the global position.

To calculate the confidence of foot grounding, it is assumed in Equation (9) that one foot is grounded. Therefore, the accuracy of the proposed approach degrades in a sequence in which a subject lifts both feet off the ground for long time, such as by jumping. We confirmed that the short period of foot takeoff does not seriously affect the accuracy by the experiment on S5-F1, which included side-skip steps. This limitation will be overcome by inferring ground contact confidence from visual context and IMU data.

## Figures and Tables

**Table 1:** 3D position error (cm) on TotalCapture dataset.

	S1			S2			S3			S4			S5			Mean
	W2	A3	F3	W2	A3	R3	W2	A3	F1	W2	A3	F3	W2	F1	F3	
Mean position error (cm)																
*RGB only* [34]	52.4	90.1	22.5	33.3	22.6	27.4	51.4	26.9	24.6	50.4	53.3	56.1	57.7	37.1	43.1	43.3
*IMU only* [3]	45.0	42.7	44.2	144	63.9	8.91	34.8	72.3	62.4	42.3	221	39.4	124	32.9	81.0	70.6
$F(E_{O},E_{RoM},E_{P})$	54.4	41.7	29.4	142	63.3	12.2	33.0	68.8	68.5	42.8	224	39.2	124	28.2	78.1	70.0
$F(full,w_{p}=1)$	**19.6**	**14.8**	**11.9**	**11.5**	**9.22**	7.37	15.3	**10.1**	14.3	**15.7**	13.8	**14.6**	**14.9**	46.7	17.5	15.8
F(full)	20.2	15.6	12.2	12.2	10.2	**7.32**	**15.2**	12.5	**11.1**	16.3	**12.3**	14.7	16.0	**10.0**	**16.9**	**13.5**
Mean orientation error (degrees)																
*IMU only* [3]	9.32	8.25	9.43	8.59	8.27	12.5	6.50	6.55	10.6	7.10	8.14	9.51	6.59	8.37	11.6	8.75
F(full)	9.38	8.45	9.45	8.74	8.51	12.5	6.65	6.63	10.9	7.07	8.20	9.52	6.72	8.37	11.3	8.83

The minimum error values are shown in bold.

**Table 2:** 3D orientation error (degrees) on TotalCapture dataset.

	S1-F3	S2-R3	S3-F1	S4-F3	S5-F1	Mean
Trumble et al. [16]	9.4	9.3	13.6	11.6	10.5	10.9
Malleson et al. [25]	7.4	**3.9**	6.7	6.4	7.0	6.3
$F_{multi}\left(full\right)$	**6.25**	5.66	6.70	**6.32**	**5.91**	**6.17**

The minimum error values are shown in bold.
